# PREVALENCE AND TRAJECTORIES OF NEUROPSYCHOLOGICAL POST-COVID-19 SYMPTOMS IN INITIALLY HOSPITALIZED PATIENTS

**DOI:** 10.2340/jrm.v56.25315

**Published:** 2024-03-12

**Authors:** Simona KLINKHAMMER, Annelien A. DUITS, Janneke HORN, Arjen J. C. SLOOTER, Esmée VERWIJK, Susanne VAN SANTEN, Johanna M. A. VISSER-MEILY, Caroline M. VAN HEUGTEN, THE NENESCO STUDY GROUP

**Affiliations:** 1 School for Mental Health and Neuroscience, Faculty of Health, Medicine and Life Sciences, Department of Psychiatry and Neuropsychology, Maastricht University, Maastricht; 2 Limburg Brain Injury Center, Maastricht University, Maastricht; 3 Department of Medical Psychology, Maastricht University Medical Center, Maastricht; 4 Department of Medical Psychology, Radboud University Medical Center, Nijmegen; 5 Department of Intensive Care, Amsterdam University Medical Center, University of Amsterdam, Amsterdam; 6 Amsterdam Neuroscience, Amsterdam University Medical Center, University of Amsterdam, Amsterdam; 7 UMC Utrecht Brain Center, University Medical Center Utrecht, Utrecht; 8 Department of Intensive Care Medicine, University Medical Center Utrecht, Utrecht University, Utrecht, the Netherlands; 9 Department of Neurology, UZ Brussel and Vrije Universiteit Brussel, Brussels Health Campus, Jette, Belgium; 10 Department of Medical Psychology, Amsterdam University Medical Center, Amsterdam; 11 Department of Psychology, Brain and Cognition, University of Amsterdam, Amsterdam; 12 Department of Intensive Care Medicine, Maastricht University Medical Center, Maastricht; 13 Department of Rehabilitation, Physical Therapy Science & Sports, University Medical Center Utrecht, Utrecht; 14 Center of Excellence for Rehabilitation Medicine and De Hoogstraat Rehabilitation, University Medical Center Utrecht, Utrecht; 15 Department of Neuropsychology and Psychopharmacology, Faculty of Psychology and Neuroscience, Maastricht University, Maastricht, The Netherlands

**Keywords:** SARS-CoV-2, long COVID, post-COVID, fatigue, cognitive complaints, post-infection

## Abstract

**Objective:**

To investigate the prevalence and trajectories of post-COVID-19 neuropsychological symptoms.

**Design:**

Prospective longitudinal multicentre cohort study.

**Subjects:**

A total of 205 patients initially hospitalized with SARS-CoV-2 (COVID-19).

**Methods:**

Validated questionnaires were administered at 9 months (T1) and 15 months (T2) post-hospital discharge to assess fatigue, cognitive complaints, insomnia, anxiety, depression, and post-traumatic stress symptoms.

**Results:**

Analyses included 184 out of 205 patients. Approximately 50% experienced high cognitive complaints at T1 and T2, while severe fatigue affected 52.5% at T1 and 55.6% at T2. Clinically relevant insomnia scores were observed in 25% of patients at both time-points. Clinically relevant anxiety scores were present in 18.3% at T1 and 16.7% at T2, depression in 15.0% at T1 and 18.9% at T2, and PTSD in 12.4% at T1 and 11.8% at T2. Most symptoms remained stable, with 59.2% of patients experiencing at least 1 persistent symptom. In addition, 31.5% of patients developed delayed-onset symptoms.

**Conclusion:**

Post-COVID-19 cognitive complaints and fatigue are highly prevalent and often persist. A subgroup develops delayed symptoms. Emotional distress is limited. Screening can help identify most patients experiencing long-term problems. Future research should determine risk factors for persistent and delayed onset symptoms.

LAY ABSTRACT

Many individuals experience long-term neurological and psychological symptoms after recovering from their initial COVID-19 illness. This study examined these symptoms in 205 patients who recovered from severe COVID-19 who required hospital treatment. Patients completed questionnaires assessing symptoms 9 and 15 months after hospital discharge. More than half of the patients experienced heightened levels of fatigue and difficulties with mental functions, such as problems with memory. In addition, a quarter had trouble sleeping and a smaller group showed symptoms of anxiety, depression, or post-traumatic stress. Overall, once symptoms had developed, they tended to last. In some cases, symptoms developed with a delay. The study highlights the importance of identifying and potentially treating patients with long-term symptoms and calls for further research to understand the factors contributing to persistent and delayed-onset symptoms.

Following SARS-CoV-2 infection, the virus responsible for COVID-19, many individuals experience persistent neuropsychological symptoms that encompass a spectrum of emotional, behavioural, and cognitive manifestations (1). These symptoms include cognitive complaints, such as concentration or memory problems, fatigue, difficulty sleeping, and emotional distress, such as anxiety, depression, and post-traumatic stress. Persistent symptoms can negatively affect quality of life (QoL) and work reintegration (2), highlighting the importance of understanding their long-term trajectory for both patient care and public health.

Neuropsychological post-COVID-19 symptoms resemble those seen following other infectious diseases, such as with Coxiella Burnetii (Q-fever), Epstein-Barr virus, or the genetically similar SARS-CoV-1, but also conditions such as mild traumatic brain injury and stroke (3–7). Research has shown that these persistent symptoms can follow varying trajectories, either worsening, remitting, or persisting, depending on the initial medical condition (3–6, 8). Post-COVID-19 research has yielded mixed results, reflecting divergent trajectories for the same symptoms (9–12). Heterogeneous methodologies, such as time since discharge and sample characteristics, probably contribute to this variability. In addition, existing studies are mostly limited to the first 12 months post-hospital discharge (9–11). Furthermore, despite frequent reports of post-COVID-19 memory- and concentration problems, there is limited knowledge on the prevalence of a broader spectrum of cognitive complaints, extending beyond just memory and concentration problems (13). Existing research often relies on a single question regarding the presence or absence of memory/concentration problems (13).

Prior research has explored the prevalence and group trajectories of post-COVID-19 symptoms, but the relative frequency of specific symptom trajectories is unknown. As a result, there is a lack of knowledge on the likelihood that an individual patient’s symptoms will persist, remain absent, remit, or have a delayed onset. This knowledge is helpful in the organization of healthcare, including early identification of those at risk and targeted treatment of individuals experiencing persistent post-COVID-19 symptoms.

Therefore, the primary goals of this prospective longitudinal cohort study were to examine: (*i*) the prevalence of a broad range of neuropsychological post-COVID-19 symptoms in previously hospitalized patients; (*ii*) differences in symptom prevalence between 9 and 15 months post-hospital discharge; and (*iii*) prevalences of the following trajectories: persistent symptoms, delayed-onset symptoms, remitted symptoms, and symptom absence. A secondary goal was to explore factors associated with unfavourable trajectories.

## METHODS

### Study design

The current article is based on the NeNeSCo study, a multicentre prospective follow-up cohort study in which participants were assessed at approximately 9 (T1) and 15 (T2) months post-hospital discharge (see 14, 15).

### Participants

Patients were eligible for participation if they had been hospitalized (intensive care or general ward) during the first European wave of COVID-19 (March to June 2020), were at least 18 years old, with objective SARS-CoV-2 infection, and sufficient Dutch language skills. Patients with pre-existing cognitive impairment diagnosed before the onset of COVID-19, as indicated in medical files and stemming from congenital or acquired brain injury or neurocognitive disorder, those with contraindications to magnetic resonance imaging (MRI), severe neurological damage after hospital discharge, or physically unable to attend hospital visits were excluded from participating in the study.

### Procedure

The recruitment process involved 6 hospitals providing lists of admitted COVID-19 patients. The order of the lists was randomized. Eligibility for the study was assessed from the top of the list, inviting patients who met the criteria until the intended sample size of 200 participants was reached. Questionnaires were administered either during a hospital visit or at participants’ homes, using paper-based or online formats.

### Demographics

Patients were administered a questionnaire assessing demographic factors, such as sex, age, and education. In addition, the questionnaire covered a range of pre- and post-illness variables, including post-COVID-19 occupational changes, expectations regarding recovery, and the history of psychological care before the onset of COVID-19.

### Screening instruments

*Cognitive complaints*. The Checklist for Cognitive Consequences following Intensive Care Admission (CLC-IC) is an adapted version of the Checklist for Cognition and Emotion (CLCE-24) and comprises 10 items evaluating the presence of cognitive complaints (rated as yes/no), with a total score ranging from 0 to 10. The cut-off point (≥ 4) was determined based on the mean (*M* = 1.9, standard deviation (SD) = 1.9) of cognitive complaints in a healthy control group (16).

*Fatigue*. The Fatigue Severity Scale (FSS) is a self-report questionnaire consisting of 9 items rated on a scale from 1 to 7, with a total score between 9 and 63. Higher scores indicate higher levels of fatigue. A cut-off score ≥ 36 was used to indicate severe fatigue.

*Insomnia.* The Insomnia Severity Index (ISI) comprises 7 items, rated on a 5-point Likert scale (rated 0–4), resulting in a total score ranging from 0 to 28. Higher scores indicate more severe insomnia. A cut-off score of 10 was used to determine clinically relevant insomnia.

*Anxiety and depression*. The Hospital Anxiety and Depression Scale (HADS) consists of 2 subscales separately measuring anxiety and depression, each comprising 7 self-report items. Items are rated on a 5-point Likert scale, with total scores ranging from 0 to 21 per subscale. Higher scores indicate higher levels of depression/anxiety and a commonly used cut-off score of ≥ 8 per subscale is utilized to identify individuals with clinically relevant symptom levels.

*Post-traumatic stress disorder*. The Primary Care Post-Traumatic Stress Disorder Screen for The Diagnostic and Statistical Manual of Mental Disorders, Fifth Edition (DSM-5) is a screening tool designed to assess the presence of post-traumatic stress disorder (PTSD). It consists of 5 yes/no questions, with a total score range of 0–5. A cut-off score ≥ 3 is used to indicate probable PTSD.

*Passive coping tendencies.* These were measured with the passive coping subscale of the Utrecht Coping List (UCL). The scale consists of 7 items, with a total score ranging from 7 to 28, where higher scores indicate a greater tendency for passive coping.

*Social support.* This was measured using the Social Support List (SSL-12-I), which includes 12 self-report items and a total score ranging from 12 to 48. Higher scores indicate greater perceived social support.

*Quality of life.* QoL was assessed with the EuroQol-5D-5L (EQ-5D-5L), comprising 5 items with 5 severity options. A summary index value was obtained by applying the Dutch value set, which represents health state preferences of the Dutch general population. The summary index ranges from 0 to 1, where 1 indicates highest QoL.

All questionnaires are validated and frequently used in clinical practice and research. Cut-off scores are grounded in previous research and, except for the CLC-IC, adhere to clinical practice standards. See Table SI for a more detailed overview and psychometric properties of the instruments.

### Statistical analysis

Only patients who completed the study (i.e. data available for T1 and T2) were included in the current analysis. Furthermore, a patient’s score per screening instrument was considered if data were available for both measurement time-points. Patient characteristics of included patients and those who were excluded (due to missing data for at least 1 full measurement time-point) were compared using χ^2^ test, Fisher’s exact test, and Mann–Whitney *U* test, as applicable. In cases where single data-points (≤ 15% per patient and screening instrument) were missing, data were mean imputed. If missing data exceeded this threshold, the patient’s score on the corresponding screening instrument was disregarded from the analysis.

- A symptom was defined as a score on a screening instrument falling above the clinical cut-off score. Therefore, patients’ scores on the 6 screening instruments were categorized into: non-clinical (below cut-off) and clinical (above cut-off). The percentage of patients in the clinical-score group denoted the prevalence.
- Prevalences were compared between T1 and T2 using McNemar’s tests. Score differences on the group level were analysed by comparing continuous scores at T1 and T2 using Wilcoxon signed-rank tests.
- Trajectories were defined as the 2 scores corresponding to T1 and T2 on a screening instrument. Trajectories were classified into 4 categories: *persistent symptom,* where the score remains above the clinical cut-off at both time-points, *delayed-onset symptom,* characterized by an initially non-clinical score developing into a clinical score, *remitted symptom*, representing an initially clinical score transforming into a non-clinical score, and *absent symptom,* indicating that the score remains below clinical cut-off at both time-points. A participant’s trajectory was categorized accordingly, per screening instrument, resulting in 6 trajectories per participant. The prevalence of categories per screening instrument were illustrated using Sankey flow diagrams, which employ nodes to describe the states (clinical or non-clinical score at T1 and T2) and arcs to visualize the 4 categories.
- The distributions of screening instrument scores classified as persistent, delayed-onset, remitted, and absent symptoms were visualized in a bar graph.
- To gain insights into the magnitude of score change that led to a reclassification from non-clinical to clinical or vice versa, the increases/decreases in scores were plotted. To facilitate cross-instrument comparisons, scores from all screening instruments were transformed into percentiles (see Table SII for details).
- To explore factors associated with unfavourable trajectories (i.e. the number of screening instrument scores categorized as either persistent and delayed-onset symptoms), Pearson correlations with continuous variables were computed (i.e. UCL, SSL-12-I, and EQ-5D-5L). Subsequently, the sum of unfavourable trajectories was dichotomized into low (≤ 1) and high (> 1) and categorical demographic and illness-related variables of patients in the low and high group, compared using χ^2^ tests.

Significance was assessed at a 2-sided alpha-level of 0.05 and statistical analyses were executed using R version 4.2.2.

## RESULTS

In total, 205 patients were enrolled and 184 (89%) completed both assessments. Their characteristics are shown and compared with dropouts in Table I. There were no significant differences between the included and excluded participants.

**Table I:** Patient characteristics

Characteristics	Total group (*n* = 205)	Included (*n* = 184)	Dropouts (*n* = 21)	*p*-value
Age, years	63 (53–69)	63 (54–70)	60 (48–66)	0.120
Sex, female	62 (30.2%)	54 (29.3%)	8 (38.1%)	0.408
Education level^a^				
Low	39 (19.0%)	33 (17.9%)	6 (28.6%)	0.454
Medium	84 (41.0%)	77 (41.8%)	7 (33.3%)	
High	82 (40.0%)	74 (40.2%)	8 (38.1%)	
Received care after hospital discharge^a^				
Physical therapy	147 (72.1%)	134 (73.2%)	13 (61.9%)	0.274
Occupational therapy	55 (27.0%)	51 (27.9%)	4 (19.0%)	0.388
Rehabilitation^b^	90 (44.1%)	82 (44.8%)	8 (38.1%)	0.557
Psychology	49 (24.0%)	41 (22.4%)	8 (38.1%)	0.111
Marital status				
Married/living together	146 (71.2%)	134 (72.8%)	12 (57.1%)	0.482
In a relationship (not living together)	12 (5.9%)	10 (5.4%)	2 (9.5%)	
Single	32 (15.6%)	27 (14.7%)	5 (23.8%)	
Widow(er)	15 (7.3%)	13 (7.1%)	2 (9.5%)	
Employment situation				
Fulltime	53 (25.9%)	50 (27.2%)	3 (14.3%)	0.170
Part-time	49 (23.9%)	41 (22.3%)	8 (38.1%)	
Retired	65 (31.7%)	60 (32.6%)	5 (23.8%)	
Unemployed	7 (3.4%)	5 (2.7%)	2 (9.5%)	
Other	31 (15.1%)	28 (15.2%)	3 (14.3%)	
Hospitalization, intensive care unit	101 (49.3%)	91 (49.5%)	10 (50.0%)	0.873

Included and dropout patients did not differ significantly on any of the characteristics (all *p* > 0.05).

a Missing for *n* = 1 subjects.

b In- and out-patient rehabilitation.

### Prevalence of clinical scores

As illustrated in Table II, clinical scores were most prevalent on the FSS (T1: 52.5%; T2: 55.6%) and CLC-IC (T1: 48.6%; T2: 53.7%) at both time-points. The most frequently reported cognitive complaints were “remembering new information” (T1: 54.3% [95/175]; T2: 53.7% [94/175]), “mental fatiguability” (T1: 53.1% [93/175]; T2: 52.0% [91/175]), and “keeping up; having become slower” (T1: 50.9% [89/175]; T2: 53.1% [93/175]). Fig. S1 presents a bar graph showing the cognitive complaint distribution at both time-points.

**Table II:** Prevalence of clinical scores and changes between T1 (9 months post-hospital discharge) and T2 (15 months post-hospital discharge)

	Clinical score, *n/N (%)*		*p*-value	Median [IQR]		*p*-value
	Time-point 1^a^	Time-point 2^b^		Time-point 1^a^	Time-point 2^b^	
CLC-IC	85/175 (48.6%)	94/175 (53.7%)	0.137	3 [1–7]	4 [1–7]	0.100
Fatigue Severity Scale	93/178 (52.5%)	99/178 (55.6%)	0.345	36 [26–49]	39 [26–48]	0.647
Insomnia Severity Index	46/179 (25.7%)	45/179 (25.1%)	1.000	5 [2–11]	5 [2–11]	0.751
Hospital Anxiety and Depression Scale-Anxiety	33/180 (18.3%)	30/180 (16.7%)	0.629	2 [1–6]	3 [1–6]	0.562
Hospital Anxiety and Depression Scale-Depression	27/180 (15.0%)	34/180 (18.9%)	0.143	2 [1–5]	3 [1–6]	0.007*
Primary Care Post-Traumatic Stress Disorder Screen for DSM-5	22/178 (12.4%)	21/178 (11.8%)	1.000	0 [0–1]	0 [0–1]	0.873

A clinical score is defined as a score above the widely accepted cut-off for the corresponding screening instrument. The *p*-values correspond to McNemar’s for differences in percentages of clinical scores at time-point 1 (T1) and T2 and to Wilcoxon signed-rank tests for differences in continuous scores between T1 and T2.

a 9 months post-hospital discharge;

b 15 months post-hospital discharge.

CLC-IC: Checklist for Cognitive Consequences following Intensive Care Admission; IQR: interquartile range.

* Significant at alpha 0.05.

### Change over time

The percentage of individuals in the clinical-score group did not differ significantly between T1 and T2 for any of the screening instruments (see Table II). Continuous HADS-Depression scores increased significantly from T1 to T2 (*p* = 0.007). However, medians and upper ends of the interquartile range (IQR) of both time-points remained below the clinical cut-off.

### Trajectories

**Fig. 1:**
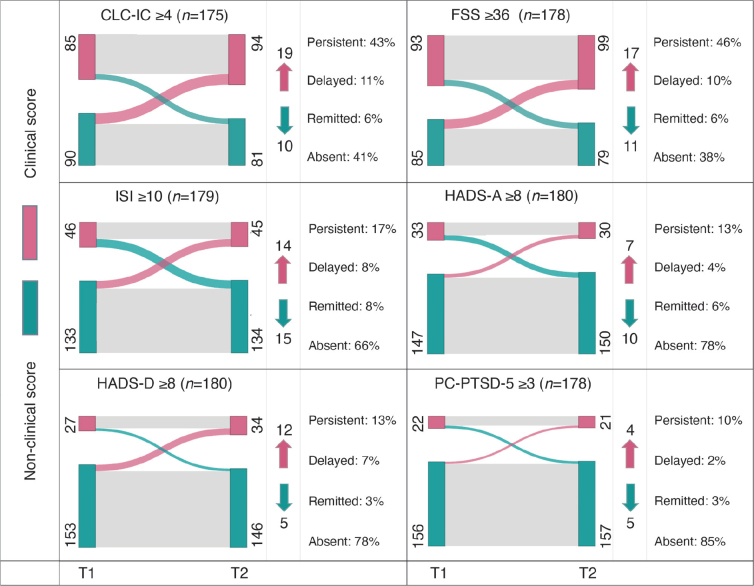
Sankey flow diagrams visualizing the prevalence of scores classified as: (A) persistent, (B) delayed onset, (C) remitted, and (D) absent symptoms per screening instrument. CLC-IC: Checklist for Cognitive Consequences following Intensive Care Admission; FSS: Fatigue Severity Scale; ISI: Insomnia Severity Index; HADS-A: Hospital Anxiety and Depression Scale – Anxiety subscale; HADS-D: Hospital Anxiety and Depression Scale – Depression subscale; PC-PTSD-5: Primary Care Post-Traumatic Stress Disorder Screen for DSM-5; T1: time-point 1 (9 months post-hospital discharge), T2: time-point 2 (15 months post-hospital discharge); *n*: Total number of patients included per measurement construct. *Red* nodes correspond to the number of patients whose score was classified as clinical (score above the cut-off), at T1 on the left and T2 on the right. Conversely, *green* nodes represent patients with non-clinical scores (score below the cut-off). The arcs in the diagram illustrate trajectories: *grey* arcs signify no change in score classification, typical of persistent or absent symptoms; red arcs indicate an increase of scores classified as delayed onset symptoms, and *green* arcs depict decreasing scores classified as remitted symptoms. Numbers indicate the count of patients in each category, with *arrows* illustrating the number of patients who transitioned from one category to another, where *red* indicates the delayed onset of new clinical symptoms, and *green* represents remission of such.

As shown in Fig. 1, scores mostly remained stable within their category, either classified as persistent or absent symptom, in 83.4% [146/175] for CLC-IC, 84.3% [150/178] for FSS, 83.8% [150/179] for ISI, 90.6% [163/180] for HADS-Anxiety, 90.6% [163/180] HADS-Depression, and for PC-PTSD-5 94.9% [169/178]. Scores were classified as delayed onset symptoms in 10.9% [19/175] for CLC-IC, 9.6% [17/178] for FSS, 7.8% [14/179] for ISI, 3.9% [7/180] for HADS-Anxiety, 6.7% [12/180] for HADS-Depression, and 2.2% [4/178] for PC-PTSD.

### Distribution of scores classified as persistent, delayed-onset, remitted, and absent symptoms

Among all patients, 31.5% [58/184] showed an increase in scores classified as delayed onset symptom on at least 1 screening instrument. Mostly, scores on only 1 instrument fell under delayed onset symptom category (43.0% [44/53]). Scores on at least 1 instrument were classified as persistent symptoms in 59.2% [109/184] of patients. Fig. S2 shows more detail.

### Score change in delayed-onset and remitting symptoms

**Fig. 2:**
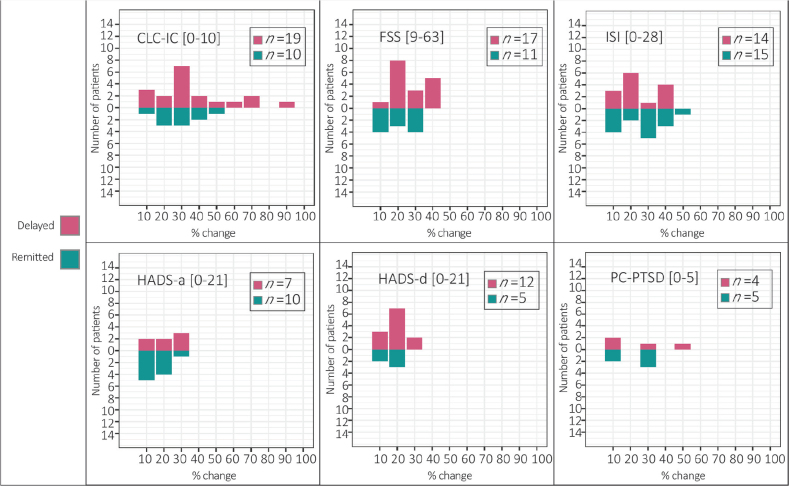
Magnitude of score change between T1 (9 months post-hospital discharge) and T2 (15 months post-hospital discharge) reported by patients who had at least 1 score classified as (A) delayed onset or (B) remitted symptom. CLC-IC: Cognitive Complaints following Intensive Care Admission, FSS: Fatigue Severity Scale, ISI: Insomnia Severity Index, HADS-a: Hospital Anxiety and Depression Scale – anxiety subscale, HADS-d: Hospital Anxiety and Depression Scale – depression subscale, PC-PTSD: Primary Care Post Traumatic Stress Disorder Screen for DSM-5, *n*: number of individuals per group. Note: The figure displays percentages of score change between T1 (9 months post-hospital discharge) and T2 (15 months post-hospital discharge) per screening instrument and the corresponding number of patients to whom it applies. Delayed onset symptoms (i.e. below the clinical cut-off at T1 but above cut-off at T2) are displayed in *red* and remitted symptoms (i.e. above the clinical cut-off at T1 and below cut-off at T2) in *green*.

The percentage of score-change causing a reclassification of into the clinical or non-clinical group (i.e. delayed onset or remitting symptom) can be seen in Fig. 2. Across all screening instruments, scores increased between 10% and 90%, with a mean increase of 30% from T1 to T2.

### Unfavourable trajectories

**Fig. 3:**
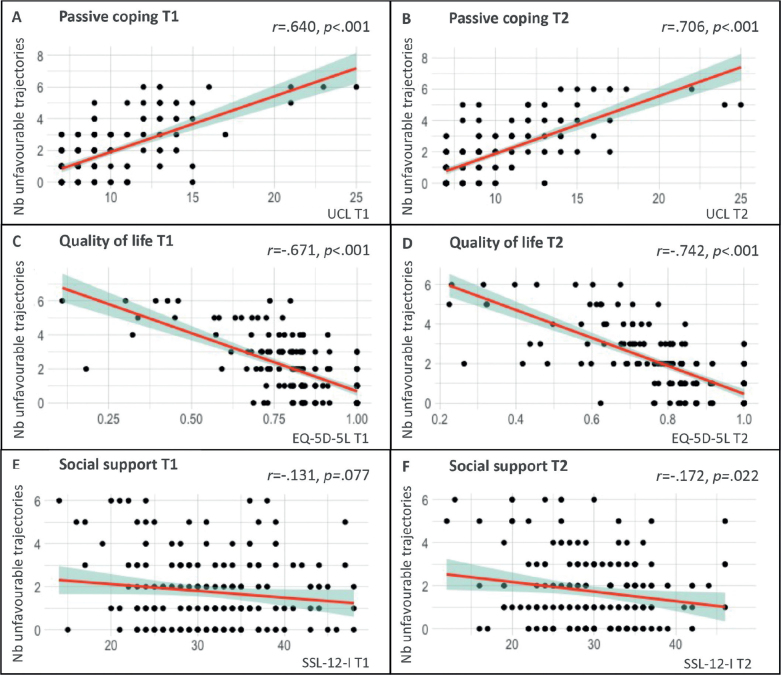
Correlations between the number of unfavourable symptom trajectories (persistent and delayed-onset symptoms) per patient and (A & B) passive coping, (C & D) quality of life, and (E & F) social support at 9 months (T1) and 15 months (T2) after hospital discharge. T1: time-point 1 (9 months post-hospital discharge); T2: time-point 2 (15 months post-hospital discharge); Nb unfavourable trajectories: number of unfavourable trajectories; UCL: Utrecht Coping List – passive coping subscale; EQ-5D-5L: EuroQol 5D-5L; SSL-12-I: Social Support List (12-item version); *r:* Pearson correlation coefficient. Note: y-axis comprises the number of unfavourable symptom trajectories (persistent and delayed-onset symptoms) per patient, while the x-axes illustrate the corresponding scores on the 3 psychosocial variables at either time-point 1 or time-point 2. Patients may exhibit a range of unfavourable trajectories, with a minimum of 0 (indicating the absence of persistent or delayed-onset symptoms on all screening instruments) and a maximum of 6 (signifying the presence of persistent or delayed-onset symptoms across all screening instruments).

A higher number of unfavourable trajectories correlated significantly with higher tendencies for passive coping and lower QoL at both time-points (all *p* < 0.001), as well as less social support at T2 (*p* = 0.02) (see Fig. 3). The analyses were rerun under exclusion of outliers (mean ± 1.96*SD). This did not change the interpretation of results.

Overall, 49.5% (91/184) of patients exhibited more than 1 unfavourable symptom trajectory. This means that their scores fell into the categories of persistent or delayed onset symptom on at least 2 out of the 6 screening instruments. In comparison with patients with a low number of unfavourable trajectories, those with more unfavourable trajectories were more frequently female (36% vs 23%; *p* = 0.042), differed in post-COVID-19 employment status (22% vs 9% on sick leave or occupationally disabled; *p* = 0.026), reported that their employment situation changed since their COVID-19 hospitalization (43% vs 22%; *p* = 0.002), received a greater amount of care after hospital discharge (physical therapy, occupational therapy, rehabilitation, psychological care; all *p* < 0.05), more frequently anticipated experiencing persistent symptoms (mild and severe, both *p* < 0.001), and reported a higher prevalence of pre-COVID-19 psychological care (37% vs 15%; *p* < 0.001). For more detail see Table SIII.

## DISCUSSION

This study explored the prevalences and trajectories of neuropsychological post-COVID-19 symptoms in previously hospitalized patients at 9 and 15 months post-discharge. The results show that over half of patients reported severe fatigue at both time-points (52.5% at 9 months and 55.6% at 15 months), and approximately half reported a high number of cognitive complaints (48.6% at 9 months and 53.7% at 15 months). Insomnia symptoms affected approximately a quarter of patients (25.7% after 9 months and 25.1% after 15 months). A smaller group exhibited symptoms of anxiety (18.3% after 9 months and 16.7% after 15 months), depression (15.0% after 9 months and 18.9% after 15 months), and PTSD (12.4% after 9 months and 11.8% after 15 months). At the group level, symptoms remained stable, with those observed at the 9-month mark persisting, while subthreshold symptoms remained absent (3). On an individual patient level, 31.5% experienced the delayed onset of at least 1 symptom (4). Unfavourable symptom trajectories correlated with a greater tendency for passive coping, lower perceived social support (at T2), and lower QoL. Patients with a low and high number of unfavourable symptom trajectories differed on several demographic and illness-related variables.

### Prevalence of neuropsychological post-COVID-19 symptoms and differences in prevalences between 9 and 12 months post-hospital discharge

These findings align with existing literature, showing a high prevalence of fatigue and cognitive complaints, with comparatively low emotional distress levels (anxiety, depression, and PTSD) (1, 17, 18). Emotional distress prevalences were lower compared with post-hospitalization rates of other viral infections, such as Middle East respiratory syndrome (MERS) and similar to pre-COVID-19 general population levels (19, 20). In contrast, insomnia complaints were more common than in the pre-pandemic general population (21).

The literature on post-COVID-19 symptom development is inconsistent. Previous fatigue studies reported varying results: symptom decreases (10, 22), stable symptoms (23), or increasing symptoms over time (9, 24). Anxiety was found to decrease in the first 12 months after hospitalization, but depression results were mixed (11, 12). Variability may be due to differences in follow-up periods and post-acute treatments, with symptoms eventually stabilizing and no observed change in the current study. Some studies assessed fatigue using a single binary question yielding potentially different interpretations and results (10, 22, 25). Importantly, post-COVID-19 fatigue has both mental and physical components, while our study’s questionnaire (i.e. FSS) emphasizes the physical component (26). Future research should assess generalizability to mental fatigue. Consistent with our findings showing high prevalence of persistent fatigue, a study of individuals surviving an infection with the genetically similar SARS-CoV observed fatigue persisting for up to 4 years after the initial illness (7).

Regarding anxiety, depression, and insomnia, contextual factors, such as country-specific governmental restrictions, though not formally studied, probably influenced these measures, contributing to inconclusive results. For example, the strictest COVID-19 lockdown in the Netherlands coincided with our second measurement and a peak in depressive symptoms in the general population (27, 28). This peak may explain the observed increase in continuous depression scores, which, on average, remained below the clinical threshold. Similarly, insomnia symptoms were elevated during lockdown compared with pre-pandemic levels, with a meta-analysis linking the pandemic to higher rates of subthreshold insomnia (29, 30).

Previous studies mostly limited cognitive complaints to memory and concentration difficulties (13). The current study reveals a broader spectrum of, often persisting, memory, mental fatigue, and cognitive slowness complaints. This study examined the trajectory of cognitive complaints in a sample not selected based on complaint experience, but the results align with studies that recruited patients with persistent complaints (31). It is crucial to distinguish cognitive complaints from dysfunction, as previous research has demonstrated a discrepancy between self-reported problems and cognitive test performance (15, 32). Consistently, we previously observed cognitive complaints exceeding dysfunction in this sample (15). Notably, psychological symptoms, such as depression, are often associated with underestimated cognitive functions (32).

### Prevalence of persistent symptoms, delayed-onset symptoms, remitted symptoms, and symptom absence

The observed trajectories in this study reflect patterns following potentially traumatic events, as described by Bonanno (33). Most individuals experience minimal disruption following such events, which they term “resilience”, with differences in trajectories often attributed to coping styles. This fits our correlation between unfavourable symptom trajectories and passive coping tendencies (34). While the emotional distress trajectories identified in the current study align with this profile, cognitive complaints and fatigue predominantly persist. This could stem from differences in psychological and biological factors contributing to symptom development, affecting the efficacy of coping strategies. Percentages of individuals with persistent, delayed onset, remitted, and absent symptoms of anxiety and depression in our severely ill population resemble those of the broader post-COVID-19 population (35).

The current study indicates that, once a symptom emerges, it tends to persist, with over half of the patients experiencing at least 1 persistent symptom. Approximately 1 in 3 individuals developed, typically 1, delayed-onset symptom. A comprehensive symptom screening post-hospital discharge would identify many patients with long-term problems, but those who develop symptoms with a delay would go unnoticed, potentially leading to a lack of treatment. Elevated occurrences of unfavourable trajectories were observed in females, patients with a history of pre-COVID-19 psychological care, those currently on sick leave or occupationally disabled, recipients of extended post-hospital discharge care, and individuals anticipating persistent symptoms. While this suggests a distinction in illness severity, the lack of difference in ICU admission does not affirm this. Future research is needed to formally study factors predisposing patients to persistent and delayed onset symptoms. Extending the follow-up period further would provide valuable insights.

The literature univocally shows that neurological recovery, driven by increased plasticity, significantly, slows down after the initial 6 months, transitioning into a chronic stage (36). This study’s notable symptom changes prompt consideration of non-biological factors. Coping, as previously mentioned, has been proposed as an influential factor, and the current study confirms its association with unfavourable symptom trajectories (34). Alternatively, resolving physical symptoms may alleviate neuropsychological symptoms. Delayed onset symptoms might stem from heightened symptom awareness due to increasing post-illness life demands (e.g. return to work) or lifted governmental restrictions (e.g. more social interactions). Persistent inflammation or chronic stress could also cause delayed symptoms (37).

Importantly, not all clinically relevant scores may be perceived as burdensome symptoms, and completing a questionnaire does not necessarily signify individuals seeking help for symptoms. Changes in scores leading to a change in symptom classification may not always be experienced as meaningful by the patients. People who score just below the cut-off at one time and just above the cut-off the next time may not have significantly different experiences. Small variations in score may also reflect common self-report fluctuations (38). Consequently, not all patients with persistent or delayed-onset symptoms may require treatment. Nonetheless, considering a mean score increase of 30% and a maximum score increase of 90%, a meaningful change can be assumed in some cases. Furthermore, the correlation between unfavourable symptom trajectories and reduced QoL suggests that symptoms do impact well-being. Future research should further explore the presence and extent of perceived symptom burden.

While most patients received physical therapy, psychological care was less common after hospital discharge (22.4% of our sample, see Table I). There may be need for additional care to address the often-persistent post-COVID-19 symptoms. Cognitive behavioural therapy, regardless of symptom origin, has demonstrated promise in alleviating the burden associated with post-COVID-19 symptoms, including severe fatigue (39). Given their potential impact on patients’ QoL and ability to return to work, ensuring adequate treatment and support for individuals with persistent symptoms is paramount (39).

Persistent post-COVID-19 symptoms are widespread and also impact non-hospitalized patients, who typically experience a milder disease course. Despite potential differences in biological contributors, psychological and social factors may similarly apply across the disease severity spectrum. A SARS-CoV-2 infection, especially during pandemic-times, may have been perceived as traumatic, irrespective of disease severity. While some individuals appear resilient to potential trauma, others develop symptoms. Factors such as personality, coping mechanisms, demographics, and social and economic resources were identified as influencing resilience (40). This aligns with our observed association between unfavourable symptom trajectories, coping tendencies, and social support.

### Strengths and limitations

This study provides a comprehensive overview of neuropsychological post-COVID-19 symptoms using validated questionnaires and an extended follow-up period. It minimizes potential bias by conducting measurements before widespread awareness of these symptoms. Inclusion of a non-preselected sample enhances generalizability and a multicentre recruitment approach augments sample diversity. Focusing on hospitalized patients may limit generalizability to non-hospitalized individuals. The rapid onset of the pandemic makes pre-COVID-19 baseline levels unclear. The absence of a non-COVID-19 control group makes it challenging to isolate effects attributed to the illness. Lastly, while symptom prevalence has been measured, their direct impact on symptom burden remains uncertain.

### Conclusion

Most severely ill COVID-19 patients will experience at least 1 persistent neuropsychological symptom, primarily cognitive complaints or fatigue. Typically, symptoms are present at 9 months post hospital discharge and persist, with some patients developing symptoms later. A comprehensive post-hospital screening could identify most patients with long-term neuropsychological problems. Identifying risk factors for persistent symptoms and delayed symptoms is a priority for future research. The need for care will depend on individual symptom burden. Furthermore, delayed effects of severe COVID-19 should be considered when patients present in primary care settings with cognitive complaints and/or fatigue.

## Supplementary Material

PREVALENCE AND TRAJECTORIES OF NEUROPSYCHOLOGICAL POST-COVID-19 SYMPTOMS IN INITIALLY HOSPITALIZED PATIENTS

PREVALENCE AND TRAJECTORIES OF NEUROPSYCHOLOGICAL POST-COVID-19 SYMPTOMS IN INITIALLY HOSPITALIZED PATIENTS

## Trial details

The study was pre-registered (ClinicalTrials.gov: NCT04745611).
