# Supplementary material for: PREVALENCE AND TRAJECTORIES OF NEUROPSYCHOLOGICAL POST-COVID-19 SYMPTOMS IN INITIALLY HOSPITALIZED PATIENTS
Source: J Rehabil Med. 2024 Mar 12;56:25315. doi: 10.2340/jrm.v56.25315 (PMC10949081; doi:10.2340/jrm.v56.25315)

Supplementary material has been published as submitted. It has not been copyedited, typeset or checked for scientific content by Journal of Rehabilitation Medicine

Fig. S1. The percentage of cognitive complaints reported at T1 and T2. Items described from left to right: 1. Organizing=difficulty with planning or organizing activities. 2. Flexibility=difficulty with flexibility. 3. Multitasking=difficulty doing to things at once. 4. Initiative=difficulty taking initiative. 5. Talking=difficulty speaking or writing. 6. Old information=difficulty remembering old information. 7. Attention=difficulty attending to things. 8. Keeping up=difficulty keeping up and having become slower. 9. Mental fatigue=experiencing frequent and rapid fatigue from mental activities. 10. New information=difficulty remembering new information. *Indicates significant differences at alpha 0.05, as assessed with McNemar's tests.

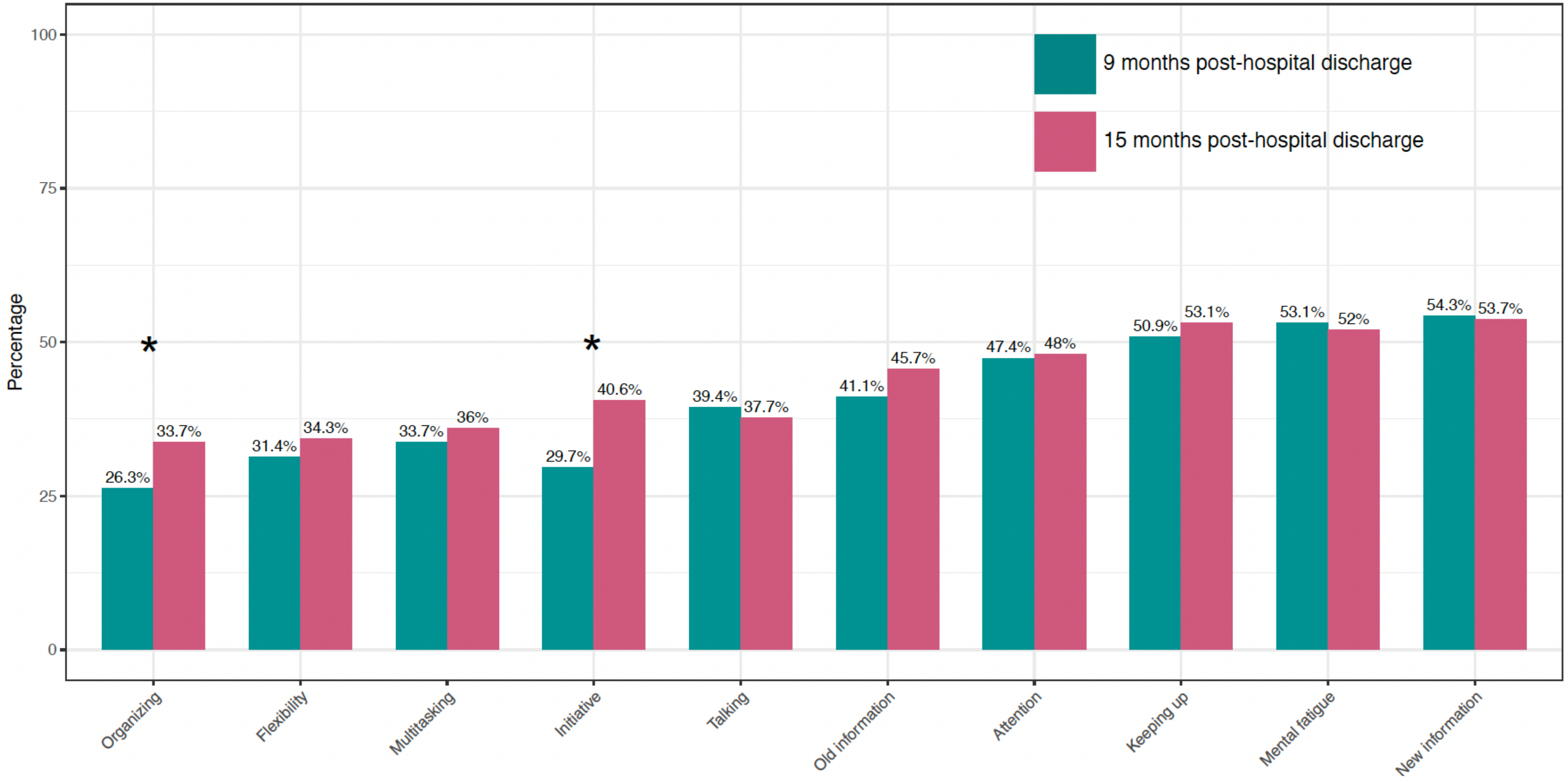

Fig. S2. The distributions of neuropsychological screening instrument scores categorized as absent, persistent, remitted, and delayed onset symptoms. The figure illustrates the four trajectory categories (absent, persistent, remitted, and delayed onset symptoms). Each patient's trajectory on each of the six screening instruments was assigned to one of these categories, resulting in six trajectories per patient. The percentages represent the distribution of screening instruments within each category for each patient. For example, 20.1% of patients are classified as having all six trajectories classified as absent symptoms, hence they had no clinical symptom at either timepoint. Further, 2.7% of patients are classified as having persistent symptoms on all six instruments.

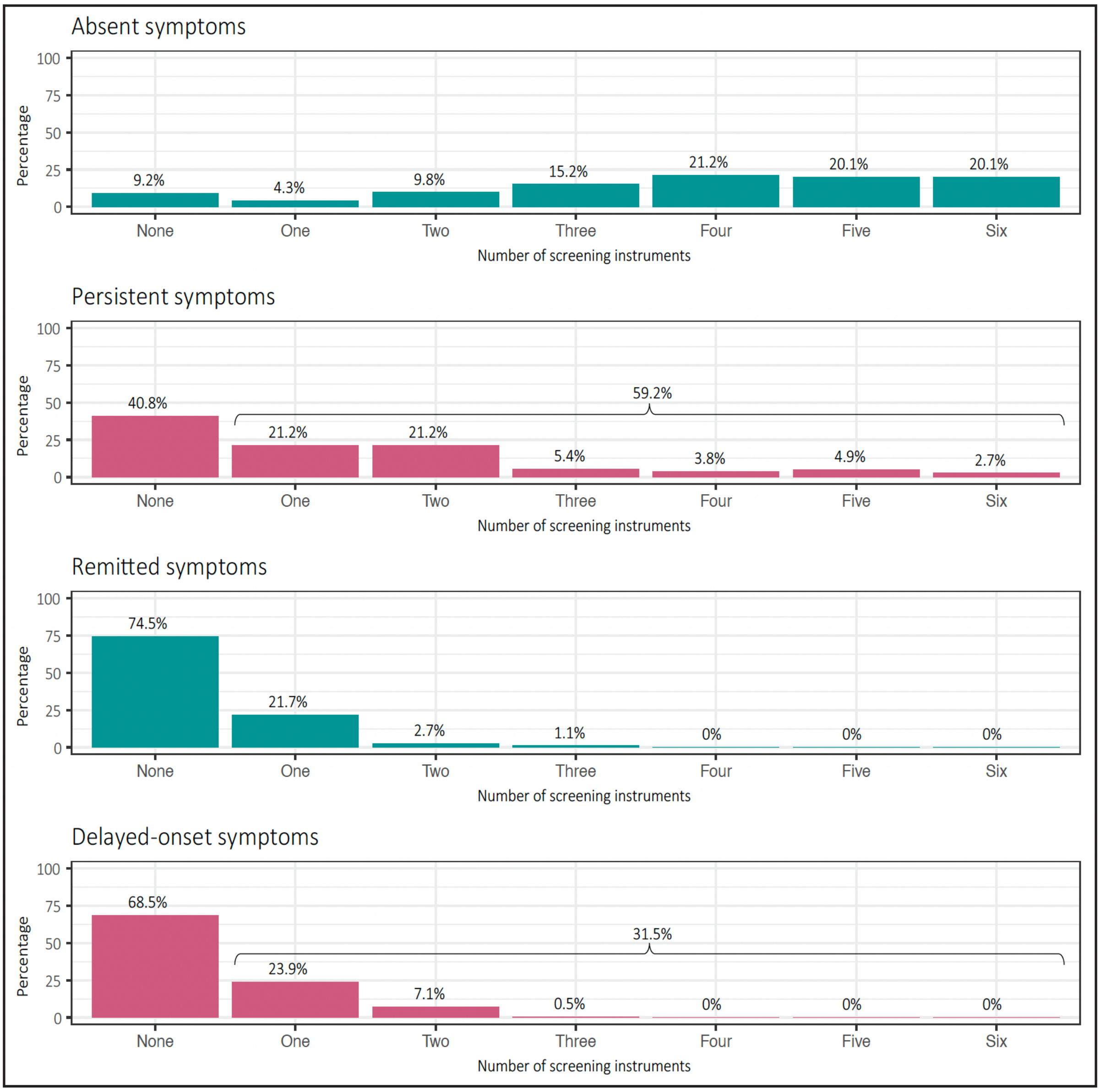

Supplement: PREVALENCE AND TRAJECTORIES OF NEUROPSYCHOLOGICAL POST-COVID-19 SYMPTOMS IN INITIALLY HOSPITALIZED PATIENTS [file JRM-56-25315-s1.pdf]
