# Supplementary material for: PREVALENCE AND TRAJECTORIES OF NEUROPSYCHOLOGICAL POST-COVID-19 SYMPTOMS IN INITIALLY HOSPITALIZED PATIENTS
Source: J Rehabil Med. 2024 Mar 12;56:25315. doi: 10.2340/jrm.v56.25315 (PMC10949081; doi:10.2340/jrm.v56.25315)
Supplement: PREVALENCE AND TRAJECTORIES OF NEUROPSYCHOLOGICAL POST-COVID-19 SYMPTOMS IN INITIALLY HOSPITALIZED PATIENTS [file JRM-56-25315-s2.pdf]

Supplementary material has been published as submitted. It has not been copyedited, typeset or checked for scientific content by Journal of Rehabilitation Medicine

Table SI. **Screening instrument interpretation and psychometric properties**

Questionnaire	Score for analysis (min-max)	Interpretation (Higher score)	Cut-off score	Cronbach alpha	Test-retest reliability	Sensitivity; specificity
Checklist for Cognitive Consequences following Intensive Care Admission (CLC-IC)[a] (1)	Number of complaints (0-10)	More complaints	≥4 (2)	0.81 (1)		
Fatigue Severity Scale (FSS) (3)	Total score (9-63)	Greater fatigue	≥36 (4)	0.96 (5)	0.74-0.86 (5)	
Insomnia Severity Index (ISI) (6)	Total score (0-38)	More severe insomnia	≥10	0.74-0.91 (7, 8)		0.86; 0.88 (7)
Hospital Anxiety and Depression Scale (HADS) - Anxiety (9)	Total subscale score (0-21)	More anxiety	>7	0.68-0.93 (10)		0.80; 0.80 (10)
Hospital Anxiety and Depression Scale (HADS) - Depression (9)	Total subscale score (0-21)	More depression	>7	0.67-0.90 (10)		0.80; 0.80 (10)
Primary Care Post-Traumatic Stress Disorder (PC-PTSD-5) (11)	Number of symptoms (0-5)	More symptoms	≥ 3	0.80 (12)		0.78; 0.87 (13)
Utrecht Coping List (UCL) – passive coping subscale (14)	Total score (7-28)	More passive coping	NA	0.73 (15)	0.87 (15)	
Utrecht Scale for Evaluation of Rehabilitation-Participation (USER-P) – restrictions subscale (16)	Total subscale score (0-100)	Fewer restrictions	NA	0.90-0.91 (17, 18)		
EuroQol-5D-5L (EQ-5D-5L) (19)	Weighted health state index (0-1)	Greater health	NA	[b]		

[a]The CLC-IC is an adapted version of the Checklist for Cognition and Emotion (CLCE-24).

[b] Internal consistency is less relevant for this questionnaire but a recent systematic review reported excellent psychometric properties across a range of populations. (20)

References:

1. Van Heugten C, Rasquin S, Winkens I, Beusmans G, Verhey F. Checklist for cognitive and emotional consequences following stroke (CLCE-24): development, usability and quality of the self-report version. Clin Neurol Neurosurg 2007; 109: 257-262. DOI: 10.1016/j.clineuro.2006.10.002.
2. van Rijsbergen MW, Mark RE, de Kort PL, Sitskoorn MM. Prevalence and profile of poststroke subjective cognitive complaints. J Stroke Cerebrovasc Dis 2015; 24: 1823-1831. DOI: 10.1016/j.jstrokecerebrovasdis.2015.04.017.
3. Krupp LB, LaRocca NG, Muir-Nash J, Steinberg AD. The fatigue severity scale: application to patients with multiple sclerosis and systemic lupus erythematosus. Arch Neurol 1989; 46: 1121-1123.
4. Shirley Ryan Ability Lab. Fatigue Severity Scale. 2016 [cited 2021 25.03]; Available from: https://www.sralab.org/rehabilitation-measures/fatigue-severity-scale.
5. Rosa K, Fu M, Gilles L, Cerri K, Peeters M, Bubb J, et al. Validation of the Fatigue Severity Scale in chronic hepatitis C. Health and quality of life outcomes 2014; 12: 1-12.

6. Morin CM. Insomnia: Psychological assessment and management: Guilford press; 1993.
7. Morin CM, Belleville G, Bélanger L, Ivers H. The Insomnia Severity Index: psychometric indicators to detect insomnia cases and evaluate treatment response. Sleep 2011; 34: 601-608.
8. Bastien CH, Vallières A, Morin CM. Validation of the Insomnia Severity Index as an outcome measure for insomnia research. Sleep Med 2001; 2: 297-307. DOI: 10.1016/s1389-9457(00)00065-4.
9. Zigmond AS, Snaith RP. The hospital anxiety and depression scale. Acta Psychiatr Scand 1983; 67: 361-370. DOI: 10.1111/j.1600-0447.1983.tb09716.x.
10. Bjelland I, Dahl A, Haug T, Neckelmann D. The validity of the Hospital Anxiety and Depression Scale: an updated literature review. J Psychosom Res 2002; 52:69-77.
11. Prins A, Ouimette P, Kimerling R, Cameron RP, Hugelshofer DS, Shaw-Hegwer J, et al. The primary care PTSD screen (PC-PTSD): development and operating characteristics. Primary care psychiatry 2003; 9: 9-14. DOI: 10.1185/135525703125002360.
12. Lathan EC, Petri JM, Haynes T, Sonu SC, Mekawi Y, Michopoulos V, et al. Evaluating the Performance of the Primary Care Posttraumatic Stress Disorder Screen for DSM-5 (PC-PTSD-5) in a Trauma-Exposed, Socioeconomically Vulnerable Patient Population. J Clin Psychol Med Settings 2023: 1-13.
13. Prins A, Bovin MJ, Smolenski DJ, Marx BP, Kimerling R, Jenkins-Guarnieri MA, et al. The primary care PTSD screen for DSM-5 (PC-PTSD-5): development and evaluation within a veteran primary care sample. J Gen Intern Med 2016; 31: 1206-1211.
14. Schreurs P, Van de Willige G, Brosschot J, Tellegen B, Graus G. Handleiding utrechtse coping lijst UCL (herziene versie). 1993. Available from: https://bsw.ugent.be/VVGP/UCL.pdf.
15. Turner H, Bryant-Waugh R, Peveler R, Bucks RS. A psychometric evaluation of an English version of the Utrecht Coping List. European Eating Disorders Review 2012; 20: 339-342.
16. Van Der Zee CH, Visser-Meily JM, Lindeman E, Jaap Kappelle L, Post MW. Participation in the chronic phase of stroke. Top Stroke Rehabil 2013; 20: 52-61. DOI: 10.1310/tsr2001-52.
17. Post MW, van der Zee CH, Hennink J, Schafrat CG, Visser-Meily JM, van Berlekom SB. Validity of the utrecht scale for evaluation of rehabilitation-participation. Disabil Rehabil 2012; 34: 478-485.
18. de Graaf JA, Volkers EJ, Schepers VP, Visser-Meily JM, Post MW. Validity of the Utrecht scale for evaluation of rehabilitation-participation restrictions scale in a hospital-based stroke population 3 months after stroke. Top Stroke Rehabil 2022; 29: 516-525.
19. Herdman M, Gudex C, Lloyd A, Janssen M, Kind P, Parkin D, et al. Development and preliminary testing of the new five-level version of EQ-5D (EQ-5D-5L). Qual Life Res 2011; 20: 1727-1736. DOI: 10.1007/s11136-011-9903-x.
20. Feng Y-S, Kohlmann T, Janssen MF, Buchholz I. Psychometric properties of the EQ-5D-5L: a systematic review of the literature. Qual Life Res 2021; 30: 647-673.

Table SII. Transformation of Screening Instrument Scores into Percentiles

	CLC-IC	FSS	ISI	HADS-A	HADS-D	PC-PTSD-5
10%	1	0-6.3	0-3.7	0-3.0	0-3.0	0-1.4
20%	2	6.4-11.6	3.8-6.4	3.1-5.0	3.1-5.0	1.5-1.8
30%	3	11.7-16.9	6.5-9.1	5.1-7.0	5.1-7.0	1.9-2.2
40%	4	17.0-22.2	9.2-11.8	7.1-9.0	7.1-9.0	2.3-2.6
50%	5	22.3-27.5	11.9-14.5	9.1-11.0	9.1-11.0	2.7-3.0
60%	6	27.6-32.8	14.6-17.2	11.1-13.0	11.1-13.0	3.1-3.4
70%	7	32.9-38.1	17.3-19.9	13.1-15.0	13.1-15.0	3.5-3.8
80%	8	38.2-43.3	20.0-22.6	15.1-17.0	15.1-17.0	3.9-4.2
90%	9	43.4-48.7	22.7-25.3	17.1-19.0	17.1-19.0	4.3-4.6
100%	10	48.8-54.0	25.4-28	19.1-21.0	19.1-21.0	4.7-5.0

CLC-IC= Checklist for Cognitive Consequences following Intensive Care Admission, FSS=Fatigue Severity Scale, ISI=Insomnia Severity Index, HADS-A=Hospital Anxiety and Depression Scale – Subscale Anxiety, HADS-D=Hospital Anxiety and Depression Scale – Subscale Depression, PC-PTSD-5= Primary Care Post-Traumatic Stress Disorder Screen for DSM-5.

Note: Difference scores were computed by subtracting T2 from T1 scores. Subsequently, the resulting difference scores were transformed into percentiles.

Table SIII. Demographic comparison of patients with a high vs. low number of unfavorable symptom trajectories.

	Total N = 184		Low N = 93		High N = 91		
	n	%	*n*	%	*n*	%	*p*-value
Sex							
Male	130	70.7	72	77.4	58	63.7	.042*
Female	54	29.3	21	22.6	33	36.3	
Education level							
Low	33	17.9	14	15.1	19	20.9	.073
Medium	77	41.8	34	36.6	43	47.3	
High	74	40.2	45	48.4	29	31.9	
Employment[a]							
Fulltime or half days	91	49.5	54	58.1	37	40.7	.026*
Retired	60	32.6	28	30.1	32	35.2	
Unemployed	5	2.7	3	3.2	2	2.2	
Other[b]	28	15.2	8	8.6	20	22.0	
Employment change							
No	125	67.9	73	78.5	52	57.1	.002**
Yes	59	32.1	20	21.5	39	42.9	
Marital status[a]							
Married/Living together	134	72.8	66	71.0	68	74.7	.119
In a relationship (not living together)	10	5.4	8	8.6	2	2.2	
Single	27	14.7	15	16.1	12	13.2	
Widow(er)	13	7.1	4	4.3	9	9.9	
Received care after hospital discharge							
Physical therapy							
No	49	26.8	33	35.9	16	17.6	.005**
Yes	134	73.2	59	64.1	75	82.4	
Occupational therapy							
No	132	72.1	79	85.9	53	58.2	<.001***
Yes	51	27.9	13	14.1	38	41.8	
Rehabilitation[c]							
No	101	55.2	59	64.1	42	46.2	.014*
Yes	82	44.8	33	35.9	49	53.8	
Psychology							
No	142	77.6	84	91.3	58	63.7	<.001***
Yes	41	22.4	8	8.7	33	36.3	
ICU							
No	93	50.5	48	51.6	45	49.5	.769
Yes	91	49.5	45	48.4	46	50.5	
Recovery expectation[d]							
Full recovery	76	41.3	52	55.9	24	26.4	<.001***
Mild persistent symptoms	90	48.9	36	38.7	54	59.3	
Severe persistent symptoms	18	9.8	5	5.4	13	14.3	
Pre-COVID-19 psychological care[d]							
No	136	73.9	79	84.9	57	62.6	<.001***
Yes	48	26.1	14	15.1	34	37.4	

[a]Fisher's exact (at least one cell has an expected count less than 5).
[b] Out of which 60.7% (17/28) were on sick leave and 17.9% (5/28) were occupationally disabled.
[c]Includes in-clinic and out-of-clinic rehabilitation.
[d] Patients were asked to which extent they expected to recover from COVID-19.
[e] Patients self-reported whether they received any kind of psychological care in the past.

Note. The patients' scores on six screening instruments were categorized into four trajectories: persistent symptoms, delayed-onset symptoms, remitted symptoms, and absent symptoms. The first two trajectories, persistent and delayed-onset symptoms, were deemed unfavorable. Each patient could exhibit a minimum of zero and a maximum of six unfavorable trajectories, corresponding to one per screening instrument. The table presented above contrasts patients with up to one (*low*) and more than one (*high*) unfavorable trajectory.
